# Temporal Coordination in Piano Duet Networked Music Performance (NMP): Interactions Between Acoustic Transmission Latency and Musical Role Asymmetries

**DOI:** 10.3389/fpsyg.2021.707090

**Published:** 2021-09-24

**Authors:** Auriel Washburn, Matthew J. Wright, Chris Chafe, Takako Fujioka

**Affiliations:** ^1^Center for Computer Research in Music and Acoustics, Department of Music, Stanford University, Stanford, CA, United States; ^2^Wu Tsai Neurosciences Institute, Stanford University, Stanford, CA, Untied States

**Keywords:** perceptual-motor coordination, interpersonal coordination, joint action, technology-mediated interaction, acoustic transmission latency, role asymmetries, time-delayed coupling synchronization, musical turn-taking

## Abstract

Today’s audio, visual, and internet technologies allow people to interact despite physical distances, for casual conversation, group workouts, or musical performance. Musical ensemble performance is unique because interaction integrity critically depends on the timing between each performer’s actions and when their acoustic outcomes arrive. Acoustic transmission latency (ATL) between players is substantially longer for networked music performance (NMP) compared to traditional in-person spaces where musicians can easily adapt. Previous work has shown that longer ATLs slow the average tempo in ensemble performance, and that asymmetric co-actor roles and empathy-related traits affect coordination patterns in joint action. Thus, we are interested in how musicians collectively adapt to a given latency and how such adaptation patterns vary with their task-related and person-related asymmetries. Here, we examined how two pianists performed duets while hearing each other’s auditory outcomes with an ATL of 10, 20, or 40 ms. To test the hypotheses regarding task-related asymmetries, we designed duets such that pianists had: (1) a starting or joining role and (2) a similar or dissimilar musical part compared to their co-performer, with respect to pitch range and melodic contour. Results replicated previous clapping-duet findings showing that longer ATLs are associated with greater temporal asynchrony between partners and increased average tempo slowing. While co-performer asynchronies were not affected by performer role or part similarity, at the longer ATLs starting performers displayed slower tempos and smaller tempo variability than joining performers. This asymmetry of stability vs. flexibility between starters and joiners may sustain coordination, consistent with recent joint action findings. Our data also suggest that relative independence in musical parts may mitigate ATL-related challenges. Additionally, there may be a relationship between co-performer differences in empathy-related personality traits such as locus of control and coordination during performance under the influence of ATL. Incorporating the emergent coordinative dynamics between performers could help further innovation of music technologies and composition techniques for NMP.

## Introduction

Cyber-based social interaction has become ubiquitous in our society due to rapid advances in interactive technologies including video-conferencing, online social networking, and even multiplayer gaming platforms and virtual reality. This has generated a wide range of opportunities for collaborative interaction across distinct geographic locations. As with in-person human interaction, many cyber-based collaborations require individuals to effectively coordinate their actions with others. However, information transmission latencies are present at greater lengths and with increasing frequency during social interactions. This can substantially impact behavioral coordination. For instance, when auditory and visual feedback from listeners in response to a speaker is absent or delayed during videoconferencing this can disrupt conversational turn-taking behaviors (O’Conaill et al., 1993). Such effects are especially poignant in ensemble musical performance. Here the timing information about a performer’s actions is extremely critical to their co-performers’ actions, all of which have an immediate effect on the quality of musical outcomes (Goebl and Palmer, 2009). This also affects audiences’ perceptions. When a temporal offset is introduced between the video of a conductor and the audio of the ensemble performance, audiences perceive conducting quality to be lower (Meals et al., 2019). As a result, while “networked” music performance (NMP) offers intriguing opportunities for experimental music and novel compositional techniques (e.g., Chafe, 2011), it also poses significant challenges to the achievement of robust, gratifying ensemble performance.

Scarce work exists on how musicians might maintain collective temporal coordination and sustain ensemble performance during NMP. Past empirical work has, however, demonstrated that acoustic transmission latencies (ATLs) disrupt the temporal dynamics of many perceptual-motor tasks. Notably, ATLs in the action-feedback an individual receives can disrupt behavioral production of tapping, speech, and musical performance (e.g., Fairbanks and Guttman, 1958; Robinson, 1972; Gates et al., 1974; Howell et al., 1983; Finney, 1997; Finney and Warren, 2002; Pfordresher and Palmer, 2002). For example, Pfordresher and Palmer (2002) found that for a pianist playing a rhythmically isochronous melody, increases in the ATL between their keypresses and the resulting sound is associated with increased temporal variability in their playing.

### ATLs and Music Ensemble Coordination

For ensemble musical performance, the effects of ATL show more complex patterns characterizing the temporal relationships between musicians’ behaviors (Delle Monache et al., 2019; see Rottondi et al., 2016 for review). Chafe et al. (2010) asked pairs of individuals to perform a coordinated clapping task to establish an initial empirical understanding of the limitations and implications of NMP. ATLs were introduced bidirectionally between the duet partners (i.e., each performer heard their co-performers’ clapping at the same, fixed latency during a given trial). There was no pre-designated “leader” in this clapping duet like an orchestra conductor. Instead, one individual started first as a solo performer (henceforth “starter”), repeating a rhythmic pattern [“dum-(rest)-da-da”], before the other joined (henceforth “joiner”) repeating the same rhythmic pattern but in a temporally staggered manner [“da-da-dum-(rest)”]. The collective rhythm was the two unison notes (clapped by both) always interleaved with a solo note (clapped by a starter or joiner).

The researchers observed the emergence of four distinct coordinative regimes, each corresponding to a different range of ATL. Most notably, when performers exhibited stable synchronized behavior at latencies of 10–25 ms they appeared to maintain symmetrical behavioral roles. Contrastingly, the emergence of a starter-joiner dynamic for latencies between 25 and 60 ms involved distinctly different, or asymmetric roles. Different ATL ranges can therefore lead to distinct, emergent temporal relationships that can support the maintenance of ongoing coordination. The effect of a given ATL may also depend on training and the instrument being played. For example, percussionists appear able to maintain tempo when experiencing both moderate and extreme delays while other instrumentalists (e.g., harpists and flutists) are potentially more affected due to melodic and agogic constraints (Jack et al., 2018; Delle Monache et al., 2019). Past work has also revealed differences in the effects of ATL among instruments with melodic constraints (Bartlette et al., 2006), as well as variations due to instrument entropy (Rottondi et al., 2015) and reverberation (Carôt et al., 2009; Farner et al., 2009).

### Asymmetries in Musical Interaction

The “functional asymmetry” in co-actor roles observed in Chafe et al. (2010) is similar to those that often emerge over the course of general, non-musical, interpersonal interaction without explicit instruction or intention (Richardson et al., 2016). Other factors that support functional asymmetries are primarily related to (1) task structure, and (2) individual traits.

#### Task-Related Asymmetries

Individuals commonly adopt functionally asymmetric behaviors based on their distinct task roles. For example, when two people move a table together one might support the table while moving backward while the other moves forward and guides the direction. There is evidence of asymmetries in musical interaction as well. Ensemble performers often have distinct roles corresponding to their part/instrument that are understood in a hierarchical fashion (i.e., the “first” violinist is the predetermined leader of a string quartet). Timmers et al. (2014) identified a leader-follower relationship in performance timing between Viola and Violin 1 and between Violin 1 and Cello, as well as mutually adaptive relationships between Violin II and Viola and between Violin II and Cello in a professional quartet. Other studies have shown that body sway movements of the individual playing the leading melody precede those of their co-performers (Goebl and Palmer, 2009; Badino et al., 2014). Interestingly, Chang et al. (2017) found that if a leader other than Violin 1 is explicitly designated during string quartet performance then the other members’ body movements tend to follow theirs. This suggests that explicit role recognition influences the overall ensemble coordination behaviors.

Such task-related movement patterns are further associated with differences in underlying neural activities. Vanzella et al. (2019) used functional near-infrared spectroscopy (fNIRS) to identify greater activation in temporo-parietal and somatomotor regions for individuals performing the second violin part vs. the first violin part in a duet. This indicates that collective goals for ensemble performance may especially shape the behavior of the follower musician. Furthermore, a functional magnetic resonance imaging (fMRI) study of dancers revealed that the level of expertise in one role compared to the other (e.g., leader role or follower role) in couple-style dances enhanced brain activation specific to the trained role during joint hand movement coordination (Chauvigné and Brown, 2018). Thus, the two types of behavior may correspond to distinct synchronizing strategies, where a greater focus on one’s own behavioral outcomes or those of the interaction can be emphasized. Electroencephalography (EEG) hyperscanning has also revealed theta- and delta-band oscillatory coupling between musicians’ cortical activity during coordination (Lindenberger et al., 2009), and stronger alpha- and beta-band oscillatory coupling for follower to leader behavior than vice versa during guitar duet performance (Sänger et al., 2013).

In addition to explicit and predetermined roles, differences in the musical content between performers’ parts may also contribute to the emergence of asymmetries in coordination. The difference in the number of notes between players’ parts can actually affect patterns of relative adaptation between piano duet performers more than assigned leadership (Goebl and Palmer, 2009). Here, the pianist whose part had twice as many notes to play was more likely to exhibit a temporal lead, regardless of who had been assigned the “leader” role. Interestingly, this behavior may be accounted for by the widely observed “more is up” phenomenon in which larger quantitative magnitudes are associated with higher physical space (Fischer, 2012; Shaki and Fischer, 2018). Thus the performer with a greater number of notes, and larger workload, may feel hierarchically higher relative to their co-performer and more responsible for leading. Further, Loehr and Palmer (2011) observed that the melodic and harmonic complexity of the accompaniment part within a piano duet resulted in temporal grouping coinciding with the melodic structure, showing increased adaptation in the accompaniment role. Relatedly, Bishop and Goebl (2020) suggest that asymmetric leader-follower relationships can make performance more predictable for performers by decreasing variability in interpretation and increasing predictability. This potentially unconscious strategy is similar to instances of non-musical joint action where co-actors make their behavior more predictable, and consequently achieve greater coordination (Vesper et al., 2011).

#### Person-Related Asymmetries

Loehr and Palmer (2011) showed that a greater difference between partners in individual preferred tempo led to an increase in their temporal asynchronies. Pianists matched for preferred tempo achieved greater interpersonal synchrony and synchronization stability during duet performance than those who were not (Zamm et al., 2016). Wing et al. (2014) examined temporal relations between musicians in two separate professional quartets performing the same piece, finding that the two groups exhibited unique patterns of symmetry and asymmetry at the beat-to-beat timescale. Thus, functional asymmetries in temporal coordination between musicians are shaped not just by differences in musical role and the musical content of their parts, but also by the performers themselves.

Volpe et al. (2016) emphasize that coordination and leadership within music ensembles can also be related to the social dynamics between individual performers. The personality traits of each performer, including locus of control and empathy, are relevant to the emergence of co-performer asymmetries in music. In particular, individual differences may at least partially contribute to the asymmetries observed in behavioral or neurophysiological patterns. For example, during a synchronized tapping task with a virtual partner, individuals with a latent internal locus of control (i.e., perceive events to be the consequences of their own actions) showed less adaptive behavior and more leader characteristics, while those with a latent external locus of control (i.e., perceive events to be caused by external forces) adopted more of a follower role, exhibiting more frequent corrective behavior (Fairhurst et al., 2013). Further, Fairhurst et al. (2014) and Konvalinka et al. (2014) have found distinct neural activity associated with leading vs. following behavior and corresponding personality traits. For example, using fMRI during a finger tapping task with a virtual adaptive partner, Fairhurst et al. (2014) identified a correlation between internal locus of control score and the tapping interval stability in those who showed “leader” behavior (e.g., less adaptive). The score was further positively related to subjective perception of the leadership role as well. Similarly in Konvalinka et al. (2014) only individuals who showed leadership behavior during synchronized tapping exhibited alpha suppression as captured *via* EEG.

Individual differences in the perspective taking dimension of empathy also affect synchronized tapping, with higher scores linked to greater anticipation of tempo-changing metronome sequences (Pecenka and Keller, 2011). Such individual differences appear to further interact with the task asymmetry between co-performers. In our own recent piano-duet work (Washburn et al., 2019), pianists with high empathy scores exhibited greater variability in temporal coordination when the performers’ parts were melodically dissimilar as compared to when they were similar, even though duet parts were rhythmically identical. Together, these studies consistently demonstrate that a person with increased empathy or perspective taking tends to be more influenced by the activity of an external stimulus, or co-performer behavior. This point is further supported by two other studies, both requiring pianists to perform the right-hand part of a piece with a pre-recorded left-hand part (Novembre et al., 2012, 2013). During the performance, researchers used transcranial magnetic stimulation (TMS) to facilitate or inhibit excitability of the right motor cortex (thus, modulating the person’s perceptual-motor experience of the left hand). The effects of stimulation were associated with increased impairment of tempo adaptation accuracy for more empathic individuals.

### Current Study

Acoustic transmission latencies clearly influence the stable coordinative states available to co-performers. However, there is little existing evidence on how these ATLs interact with the aforementioned task and personality-related factors. Direct manipulation of ATL between co-performers may systematically influence how each type of asymmetry plays its role. In the current study we assessed this possibility by examining how two pianists, playing in separate rooms without seeing each other, adapt their coordination patterns during naturalistic, simple, rhythmic duet tasks at a given ATL. We composed eight original duets employing the same interlocking rhythmic pattern with two independent but equal parts previously studied with clapping (Chafe et al., 2010). These duets included two forms of task-related asymmetry: performer role asymmetry (starting vs. joining roles), and musical part asymmetry (similar vs. dissimilar musical parts with respect to pitch range and melodic complexity). To examine the effects of person-related asymmetry we evaluated perspective taking and locus of control. During duet performance, we introduced three ATLs (10, 20, and 40 ms), allowing us to evaluate how ATL interacts with both musical task-based and person-related asymmetries in shaping coordination. In the past decade standard internet latencies have typically ranged from 20 to 100 ms (Cáceres et al., 2008; Cáceres and Chafe, 2010), with current 5G networks producing latencies on the order of tens of milliseconds (Landström et al., 2016). The ATL values for the current study therefore fall within typical internet latencies while targeting three characteristic effects. These include a speeding up, steady maintenance, or slowing down of the average tempo for duet performance when initial performance tempo is around 90 beat-per-minute (bpm) (e.g., Farner et al., 2009; Chafe et al., 2010; Rottondi et al., 2015).

Our primary coordination measures were the magnitude and variability of note-onset asynchronies between co-performers at unison points in each duet as well as the magnitude and variability of tempo. Based on previous work, we predicted that starting performers would exhibit greater note-onset asynchrony leads, and less variability in both note-onset asynchronies and tempo. We also expected possible interactions between performer role and musical part asymmetry, with greater differences between starters and joiners in both asynchrony and tempo when the duet parts were dissimilar. We predicted that ATL would moderate these effects, with greater ATL leading to greater differences in starting vs. joining behavior. This study’s overarching goal was to provide a foundation for understanding how key aspects of the music ensemble setting shape co-performer interaction within cyber-mediated environments such as NMP.

## Materials and Methods

### Participants

We conducted a power analysis for the number of participant pairs in the current study. We based this on the Pearson correlation coefficient (*R* = 0.96) for the relationship between ATL and collective lead/lag from Chafe et al. (2010) (we refer to this measure as “cycle asynchrony,” see section “Measures and Analyses”). Using a significance level of alpha = 0.05, an intended power of 0.9, and a directional analysis without bias-correction, we obtained a sample size of *N* = 6 pairs of participants. We recruited 12 pairs of performers, satisfying the minimum sample size supported by the power analysis.

Twenty-four pianists (12 pairs) ranging from 18 to 47 years old were recruited from the Stanford University community. All had at least 4 years of piano-playing experience and all but one were active musicians, playing an instrument or singing at least 2 h a week. No one reported hearing problems relevant to their musical pursuits.^1^

Of the pairs recruited, three pairs knew each other and had played music together prior to the experiment, ranging between one and six occasions. These three pairs did not exhibit particular advantages against the ATL effects compared to the other nine pairs, or consistent outlying values for within-pair differences in the perspective taking and locus of control scores compared to the other eight pairs included in the correlation analyses. The study was approved by the Stanford University Institutional Review Board. All participants provided informed consent *via* a signed form and were paid $20/h for participating.

### Apparatus

**FIGURE 1:**
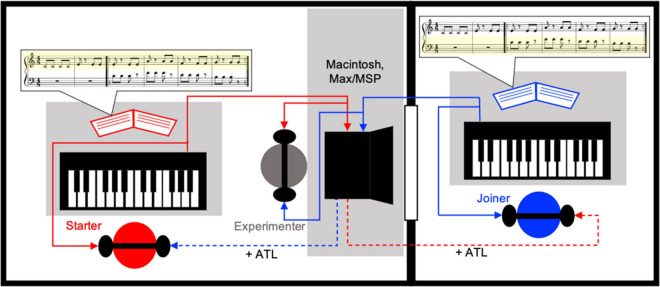
The experimental set-up. The experimenter (center, gray circle) was able to see both pianists (red and blue circles) while they were located in separate rooms and could not see each other. The keyboards were controlled by the experimenter’s Macintosh computer with Max/MSP software such that the pianists always heard their own playing at the base latency inherent within the apparatus (solid red and blue lines) but heard their partner’s playing with an additional ATL of 10, 20, or 40 ms (dashed red and blue lines) during experimental trials. In this figure the left pianist (red) is performing the starter role (playing the associated yellow highlighted score part), with the right pianist (blue) performing the joiner role (playing the associated yellow highlighted score part). All pianists played both parts of all musical compositions; this role distribution occurred for half of the trials, while for the other half the roles were reversed.

Two Yamaha Axiom-61 digital keyboards were located in two adjacent rooms, where the smaller room included sound shielding for use during EEG studies (see Figure 1). Each room was equipped with a pair of AKG K271 MKII Closed-Back headphones. We made a custom program using the Max/MSP 7.0.1 platform to not only synthesize and control all acoustic feedback but also to monitor accuracy of the performers’ keypresses and record all the keypress timing data explicitly associated with the individual notes of the duet compositions throughout the study *via* a Macintosh computer (OSX 10.9.5). Sounds recorded from the built-in OSX MIDI sound synthesizer, AU DLS Synth, were precisely triggered to create the piano timbre used throughout and the snare drum “cross-stick” timbre used for introductory metronome count-ins. The experimenter sat with this computer in the larger room. Play-back loudness was set constant regardless of the MIDI keypress velocity with pianists unable to produce changes in dynamics during performance. Note duration was fixed at 200 ms regardless of performer key-offset timing. These settings were controlled so that we could best examine the effects of the experimental manipulations of interest on temporal coordination.

As demonstrated by Wright et al. (2004), we evaluated the latency inherent to the experimental apparatus by obtaining audio recording for the following events simultaneously: (1) the acoustic sound of each keyboard keypress (captured with an AKG C 414 B-ULS microphone from approximately 5 cm distance) and (2) the resulting acoustic feedback (a piano tone emitted by the apparatus), *via* a direct electrical connection from the output of the sound generator into the input of the audio interface connected to Audacity software. From these recorded audio files we detected the instantaneous onset time of each event using a simple amplitude threshold (set to 10 times the maximum amplitude of the recorded background noise) and the rule that after one onset the amplitude must remain below the threshold for ∼2 ms before the next onset can be detected. Subtracting the keypress onset time from the corresponding piano tone onset time gives the latency for each keypress. Following outlier removal the average keypress to acoustic feedback onset for Keyboard 1 (larger room) press to Keyboard 1 audio was 33.5 ms (SD = 3.3 ms), for Keyboard 2 (smaller room) press to Keyboard 2 audio was 25.4 ms (SD = 2.0 ms). The base latency for Keyboard 1 press to Keyboard 2 audio was 27.7 ms (SD = 2.5 ms), and for Keyboard 2 press to Keyboard 1 audio was 35.3 ms (SD = 3.6 ms). Kolmogorov–Smirnov tests were used to assess whether there were significant differences between the distributions of the mean-centered latency for: (1) self-feedback at each of the two keyboards (i.e., K1-K1 vs. K2-K2), (2) self vs. other feedback at Keyboard 1 (i.e., K1-K1 vs. K2-K1), (3) self vs. other feedback at Keyboard 2 (K2-K2 vs. K1-K2), or (4) other-feedback at each of the two keyboards (i.e., K1-K2 vs. K2-K1). All tests were not significant.

Importantly, the latency differences between self-produced sound and the other keyboard sound were compatible (Keyboard 1: 33.5 vs. 35.3 ms; Keyboard 2: 25.4 vs. 27.7 ms). Performers had the opportunity to calibrate to the base latency at their own keyboard during the initial practice and test trials in the same way that musicians regularly adjust to the inherent base latency of a given environment. This means that, in our recorded timing data, the effect of the additional ATL from the partner’s keyboard sound in the experimental condition was only compared to each respective self-produced action-to-playback latency.

The Max/MSP program was used to introduce additional bidirectional ATLs of 10, 20, or 40 ms between performers during experimental trials as described below in section “Procedure.” In these trials both pianists experienced a given latency of experimentally induced delay in the acoustic outcomes of their partner’s keypresses, but not their own. Throughout every trial the Max/MSP program tracked the pianists’ performance (the MIDI inputs from the two keyboards) for accuracy in note pitch and sequence compared to the musical score to identify errors. With the introduction of ATLs we expected tempo changes, and therefore allowed all timing distortions as long as the duet collectively played the correct alternation of solo notes and “unison” notes (regardless of the timing difference in note-onset). The program also controlled the order of the ATL conditions and metronome tempo, and generated codes for events associated with experimental conditions to store these with timepoints for the data analyses.

### Stimulus and Task

**FIGURE 2:**
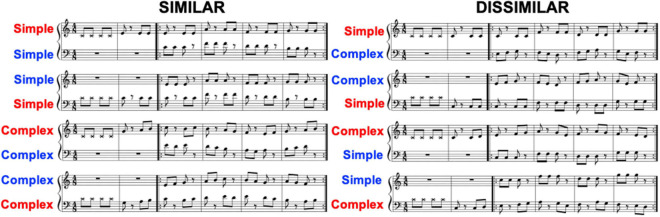
Music scores of eight piano duets composed for this study. Each duet consisted of two parts (e.g., lines) that were either *similar* or *dissimilar*, as defined by pitch range and melodic complexity. In *similar* duets (left panel) the pitch ranges of the two parts overlapped while in *dissimilar* compositions they did not (right panel). *Similar* duets also included parts that were either both melodically simple or complex, while *dissimilar* duets included one of each kind of part. For half of the duets the starting part (red labels) was placed in the top line with the joining part (blue labels) in the bottom. For the other half the line placement was reversed. Pianists had to repeat the four bars of each duet indicated by the repeat signs a total of four times. The metronome count-ins for the starting pianist were added to the starter’s line.

Eight duets in C Major were composed where each performer was meant to play either the top or bottom line (see Figure 2). All duets employed the same interlocking rhythmic pattern, with two independent but equal parts such that temporally synchronized performance results in unison just once every two notes, and the analysis of a lead/lag relationship between parts is possible (i.e., metrical phase for each part can be assessed individually) (Chafe et al., 2010). All parts were to be played with the right hand. Each duet included four full bars in which both performers played, along with a preceding pickup bar to be played by the “starting” performer. Both performers played the same rhythmic pattern consisting of three consecutive eighth notes followed by one eighth rest. However, the parts were temporally staggered such that when one player had the rest, the other player had one eighth note to play. The composition’s repeat sign meant that the players had to repeat their part four times before ending the trial at the first note of the fifth repeat.

We employed two factors to define partners’ musical part similarity for the duets (Figure 2). The first of these was the pitch range of the co-performers’ melodic parts; the two parts had either overlapping pitch ranges (sharing at least one pitch), or were separated into two distinct ranges (at least three semitones apart). In our “similar” conditions, the unison notes had an average interval of 6.16 semitones (range 3–10) while for the “dissimilar” conditions it was 14.1 semitones (5–22). The second factor contributing to musical part similarity was melodic complexity (simple vs. complex), with players assigned to the same or different types. In our melodies, “simple” ones always used three-note phrases with one-pitch repetition, while “complex” melodies had three-note phrases out of three different pitched notes, leading to a more dynamic melodic contour change over the course of the composition.

In the *similar* duets the performers’ parts were either both simple or both complex. By also requiring that each condition included one duet that started on the top line and one that started on the bottom line, four *similar* duets were generated. The *dissimilar* duets had distinct pitch ranges and always combined one melodically simple part and one melodically complex part. This resulted in eight total compositions: four *similar* and four *dissimilar*. We employed the pitch range distance and melodic complexity factors to manipulate melodic similarity between the duet parts based on previous research findings. Halpern (1984) found that musicians and non-musicians alike rated melodies least similar when they have different pitch contour and rhythm. Further, when two melodies are presented in an interleaved manner (e.g., alternate one note between the melodies), listeners can identify melodies only when their pitch ranges do not overlap (Dowling, 1973), closely related to Gestalt principles of similarity resulting in auditory streaming (Bregman, 1990). In a statistical learning study which also used alternating tones, listeners learned the statistical regularity within each melodic stream much better when the two melodies were perceptually well segregated by a contrasting grouping cue such as timbre or pitch range (Creel et al., 2004). Our duet compositions follow these principles straightforwardly. Each performer was given the opportunity to act as both the starter and joiner on each duet, for a total of 16 compositions. Duet order was organized pseudo-randomly by pair with at least one *similar* duet and at least one *dissimilar* duet occurring within both the first four compositions and the last four compositions. There were never more than three of either the *similar* or *dissimilar* duets in a row within the full presentation of the eight duets. Similarly, at least one top-line starting duet and one bottom-line starting duet occurred in both the first four duets and the last four duets and no more than three same-line starting duets ever occurred in a row. For a given duet, pianists played alternating starter/joiner roles one after another meaning that no more than two compositions in a row had the same starting vs. joining player assignment.

### Procedure

Paired participants received instructions together before being randomly assigned to separate rooms, within which each had a USB/MIDI keyboard controller and headphones (see “Apparatus” section above and Figure 1 for details). One pianist sat in the same room as the experimenter (larger room), while the other pianist was alone (smaller room). A window between the rooms allowed the experimenter to see both individuals, and vice versa, but the participants were not able to see each other when seated in front of their respective keyboards. With the headphones participants were able to hear acoustic output corresponding to their own playing, as well as their partner’s. Overall volume levels were adjusted to performers’ comfort.

Performers received binders with copies of the 16 duet compositions with the part they were to play in each highlighted for them (i.e., the starting vs. joining line). As the starter, performers were to follow the metronome tempo they heard *via* their headphones to begin the pickup measure, while as the joiner they would take tempo from the starter. Only after completing seven successful performances of a given composition would the pair move on to the next (as described below, these seven performances included one test trial without ATL, and six experimental trials with two trials at each of the three ATLs). Performers were not given any specific information about the ATL conditions but asked to play the notes of their part accurately while maintaining coordinated playing with their partner and also aiming to maintain the tempo of the metronome heard by the starting player as best as possible. A successful trial required them to play all notes of the pickup and four repeats of the subsequent four bars of a composition accurately and in the correct order, ending on the first note of what would be the fifth repeat of the composition (i.e., 52 total notes for the starting part and 49 total notes for the joining part). If any notes were missed or incorrect pitches were played the trial would abort immediately and they would start again (cued by a new metronome count-in).

Each time a new composition was presented, the performers could practice their part for a few minutes to be able to play successfully with comfort. During this practice period both performers could hear each other (at the base latency for the apparatus with no additional ATL introduced). Once they were comfortable, performers would indicate to the experimenter that they were ready to begin the recorded trials. At the beginning of each recorded trial a metronome consisting of four eighth note beats was presented exclusively to the starter. The first recorded trial following the practice period was a test trial to establish that the pair could successfully play the duet under normal acoustic conditions (i.e., with the base latency for the apparatus). The tempo of the metronome in these trials was always 90 bpm (e.g., one eighth note = 666.67 ms) where in 4/8 time the beat occurs at the eighth note level, consistent with the average tempo used in the clapping duet study by Chafe et al. (2010).

In the six experimental trials for each composition, bidirectional ATLs were introduced between performers (i.e., performers heard their own playing at the base latency but heard their partner’s playing with an additional ATL). Three different ATLs were used: 10, 20, and 40 ms. Latencies around or below 10 ms are associated with persistent anticipatory behavior between co-performers, resulting in a progressive increase in playing tempo over the course of a performance (Chafe et al., 2010). Interestingly, this range overlaps with the acoustic latencies typically experienced by performers in small chamber music ensembles, which have previously been reported as 6–9 ms (Chafe et al., 2010) or 5–10 ms (Bartlette et al., 2006).^2^ Latencies between 10 and 25 ms are found to support a high incidence of synchronous behavior between performers and stable tempo. At latencies of 25–60 ms performers begin to show either decreases in tempo or the formation of a new strategy for maintaining synchronization, namely a consistent starter-joiner dynamic. Beyond 60 ms ATL coordination generally deteriorates until performance is no longer sustainable. Each of the three ATLs employed in the current study were meant to elicit one of each of the distinct coordinative states preceding coordination deterioration.

An example of the organization of ATL and tempo conditions for the set of seven required trials associated with a single composition is provided in Table 1. The first of these trials was always the test trial (base apparatus latency with no added ATL, 90 bpm). Unlike the test trial, the six experimental trials for a given musical composition used two different tempo setups, either 84 or 96 bpm. While the average of these two tempi is 90 bpm, this variation was introduced to engage participants and maintain attention over the course of the session as it required them to adjust their internal tempo frequently between trials and respond to a given ATL rather than relying on memory. Within the six experimental trials each ATL was introduced twice and each tempo was presented three times. Presentation of the ATL and tempo conditions was organized into two blocks of three trials each. Within a three-trial block ATLs were presented pseudo-randomly such that each ATL was experienced once before any ATL was repeated. Tempo order for the six experimental trials associated with a given composition was also pseudo-random with one tempo experienced just once in the first three trials and the other experienced just once in the remaining three trials. Importantly, the tempo associated with the second presentation of a given ATL within these trials was always different from the one used for the first presentation. As noted in the “Apparatus” section above, all tempo changes during a trial were allowed as long as the duet collectively played the correct alternation of solo notes and “unison” notes (regardless of the timing difference in note-onset).

**TABLE 1:** Example ATL and tempo condition organization for the seven required trials associated with a single composition.

Trial	ATL	Tempo
1 (test)	0 ms (base)	90 bpm
2 (experimental)	20 ms	94 bpm
3 (experimental)	10 ms	94 bpm
4 (experimental)	40 ms	86 bpm
5 (experimental)	20 ms	86 bpm
6 (experimental)	40 ms	94 bpm
7 (experimental)	10 ms	86 bpm

Each successful trial took between 18 and 48 s to complete, as performances with the longer ATLs took a longer time to finish due to the collective slowing described in the results below. The six experimental trials associated with each of the 16 compositions resulted in a total of 96 trials for each pair. Following the duet-playing task, individual participants were asked to stay in their separate rooms to complete Davis’s (1980) Interpersonal Reactivity Index and the Internality, Powerful Others, and Chance (IPC) Scales (Levenson, 1981) before they were debriefed about the purpose of the study together. Each session took approximately 1.5 h.

### Measures and Analyses

Performers’ coordination behavior was examined using MIDI keypress timing for the notes to be played in unison based on the interlocking rhythmic pattern underlying all compositions (i.e., the first and third eighth note of every measure following the pickup). There were 33 of these unison points in every trial. In total, four timing measures were extracted. Two measures of collective temporal dynamics, *cycle asynchrony*, and *collective tempo* allowed comparison to previously reported effects of ATLs on rhythmic clapping (Chafe et al., 2010). Two additional timing measures, *note-onset asynchronies* and *individual tempo*, allowed us to further investigate the effect of ATLs on inter- and intrapersonal musical timing, respectively.

Questionnaire data from the *Interpersonal Reactivity Index* (Davis, 1980) and the *IPC Scales* (Levenson, 1981) were used to gather a perspective taking score and locus of control score for each performer, respectively. This allowed us to identify relationships between performer personality characteristics, performer role (i.e., starting vs. joining), and environmental ATLs in shaping temporal coordination. Details of each of these measures are described below.

#### Cycle Asynchrony

We can define a “cycle” as a single notated measure of an interlocking duet composition (shown in Figure 2). This consists of a “unison” note (both players synchronously), one player’s solo note, a second unison note, and the other player’s solo note. Each unison note supposed to be played synchronously was, in reality, associated with two individual keypress timepoints. The total amount of time disparity between performers within this one cycle would express the collective anticipation (lead) or lateness (lag) of performers with respect to each other’s playing at that moment (Chafe et al., 2010) [note that Chafe et al. (2010) referred to this measure as “collective lead/lag” and calculated it as a percentage rather than a proportion as we have here]. Effectively, this measure captures whether performers are both consistently leading or lagging each other to display acceleration or deceleration in a given cycle. For example, if the starter leads the joiner at one unison note, and the joiner leads the starter at the next then the duet is displaying acceleration. This was calculated for a pair of unison points as in Eq. 1,


(1) $$\text{lead}/\text{lag}\left(k\right)=\left(start_{\text{unison}}\left[n\right]\right)-\left(join_{\text{unison}}\left[n\right]\right)+\left(join_{\text{unison}}\left[n+1\right]\right)-\left(start_{\text{unison}}\left[n+1\right]\right)$$

where *start*_*unison*_[*n*] and *join*_*unison*_[*n*] correspond to the onset timing of the *n*-th unison note played by the starting player and joining player, respectively, while 2*k* equals to *n* (i.e., *n* is an even number). This method of evaluating asynchronies preserves the sign of the difference, thus maintaining information about the potentially dynamic relationship between performers occurring over the course of a trial.

Since a given trial would contain 16 cycles in total, the 16 lead/lag values obtained were then averaged to provide a measure of the overall acceleration or deceleration exhibited by the pair across all cycles within a trial. Each pair produced 16 trials in each of the part similarity (similar vs. dissimilar) × ATL (10, 20, and 40 ms) condition combinations. We used these trials to establish an average cycle asynchrony per pair for each part similarity × ATL condition combination. These pair averages per ATL were then averaged to identify the characteristic cycle asynchrony for each ATL condition across all pairs.

#### Collective Tempo

This measure expresses a momentary tempo estimate of the performance as it evolved over the course of a trial. First, we determined a collective unison time for each of the 33 unison points as the midpoint between player onset times. We then found the inter-unison intervals (IUIs) between the collective unison times for a given trial, resulting in a total of 32 IUIs. At the *n*-th unison note, the interval value was then expressed as a tempo value (in bpm; beat per minute) by Eq. 2,


(2) collectivetempo(n)=60/(collectiveIUI[n])

The collective tempo values for each trial were used to visualize tempo drift occurring over the course of a single performance. We averaged these tempo drift series associated with each part similarity × ATL condition combination to generate an average tempo drift series per pair for each condition combination. We then averaged these tempo drift series within condition combinations to provide a characteristic tempo drift series for each condition combination across all pairs.

#### Note-Onset Asynchronies

Note-onset asynchronies were also evaluated with respect to the performers’ starting and joining roles in a given trial. This allowed us to establish the frequency with which each player led or lagged the other within the trial, the average magnitude of temporal lead for each player when they played first at unison points (“asynchrony lead”), and the standard deviation of temporal lead at these points (“asynchrony variability”). The magnitude of a single unison point asynchrony in this context was calculated as the proportion of a beat based on the starting tempo for the given trial as


(3) $$\text{asynchrony}\left(n\right)=\left(\text{tempo}/60\right)\times \left(start_{\text{unison}}\left[n\right]-join_{\text{unison}}\left[n\right]\right)$$

Note that if this value is negative, that means that the starter played first at the unison. Thus, the negative values that occurred within a given trial were used to identify the frequency of starter leading, the average magnitude of leading, and the standard deviation of leading magnitude during a trial. For the positive values from (Eq. 3) the inverse was taken to calculate the frequency, average lead, and standard deviation of joiner-led asynchronies during a trial.

Ultimately, a single pair produced 16 trials in each of the part similarity (similar vs. dissimilar) × ATL (10, 20, and 40 ms) condition combinations. From those trials, we identified the average frequency of leading for each participant when they were assigned the starter role and when they were assigned the joiner role. We used these values to identify the within-person difference in frequency of leading for the starter vs. joiner role. This within-person difference was averaged per pair for each of the condition combinations, and then across pairs to give a characteristic within-person difference in frequency of leading for the starter vs. joiner role associated with each of the condition combinations. For each of the trial-wise measures of asynchrony lead and asynchrony variability we calculated the unique starter and joiner averages for a pair across the 16 trials in each of the part similarity × ATL condition combinations. These pair averages were ultimately used to establish the unique average asynchrony lead and asynchrony variability for starters and joiners in each of the noted condition combinations across all pairs.

The cycle asynchrony measure depicted the collective leading vs. lagging behavior exhibited by both members of a pair over the course of a “cycle” including two unison notes and two solo notes per performer. The asynchrony lead measure revealed the magnitude of leading displayed by the performer who played first at each unison timepoint. While these measures are related, cycle asynchrony can be understood as representing the mutual temporal disconnect between performers over the course of an exchange in solo behavior. Alternatively, asynchrony lead establishes the absolute temporal lead exhibited by whichever performer plays first at each unison timepoint, and allows for a comparison between starter vs. joiner behavior. Asynchrony variability also provides further opportunity to evaluate possible differences in starter vs. joiner asynchrony behavior during performance.

#### Individual Tempo

We also evaluated individual tempo through the IUIs derived for the starting and joining player separately in each trial, as in Eq. 4,


(4) playertempo(n)=60/(playerIUI[n]).

These series were used to determine the average and standard deviation of starting and joining player tempo for each trial.

For each of the average individual tempo and standard deviation of individual tempo measures we calculated the unique starter and joiner averages for a pair across the 16 trials in each of the part similarity (similar vs. dissimilar) × ATL (10, 20, and 40 ms) condition combinations. We then used these pair averages to determine the overall average individual tempo and individual tempo variability across all pairs in each condition combination.

#### Perspective Taking and Locus of Control

The Interpersonal Reactivity Index (Davis, 1980) contains a Perspective Taking Subscale with seven questions. A higher score on this subscale is indicative of more frequent perspective taking, with the maximum score being 28 and the minimum being zero. One participant did not provide responses to this index, resulting in data for a total of 23 participants for this measure.

The IPC scales (Levenson, 1981) contain the Internal Locus of Control Subscale with eight questions. The maximum score on this scale is 48 and the minimum is zero, with higher scores associated with a stronger sense of internal locus of control possessed by a person. The participant who did not provide data on the Perspective Taking Subscale also did not provide responses to this subscale, resulting in data for the same total of 23 participants for this measure.

We analyzed correlations between each of the perspective taking and locus of control scores and our behavioral measures at both individual and pair levels, as described below in section “Results.” Correlations based on individual behavioral measures included 23 data points, while those based on pairwise behavioral measures included 11 data points.

### Statistical Analyses

For all ANOVAs conducted as described in the Results Greenhouse–Geisser correction was applied for the degrees of freedom (dfs) when the assumption of sphericity was not met. Corrected dfs are reported. *Post hoc* tests were performed using either additional ANOVAs for interactions or Fisher’s least significant difference (LSD) pairwise comparisons with Bonferroni correction for main effects.

For all statistical tests, the significance level was set at alpha = 0.05. Analyses were performed using SPSS (ver. 20, IBM Inc.).

## Results

Pairs performed between 110 and 174 trials to achieve the 96 required successful experimental trials. An average of 73% (SD = 9%) of the total trials performed by each pair were deemed successful and included in our analyses.

### Cycle Asynchrony

**FIGURE 3:**
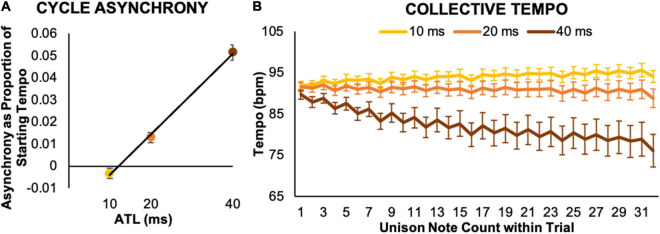
Collective temporal behaviors. **(A)** Cycle asynchrony indicates how much lead or lag performers produced in one cycle of the collective rhythmic pattern, expressed as a ratio to the initial metronome tempo. It revealed that both performers’ keypress timing preceded each other’s keypress timing at the 10 ms ATL and then lagged each other’s behavior by increasing amounts at the longer 20 and 40 ms ATLs. **(B)** Collective tempo indicates the tempo estimated at each unison note position. Over the course of a trial performers sped up in the 10 ms ATL condition, maintained a consistent tempo in the 20 ms ATL condition, and got substantially slower in the 40 ms ATL condition. Error bars show standard error.

An initial simple regression model including both ATL and part similarity (IVs: part similarity and ATL; DV: cycle asynchrony) revealed no effect of part similarity, so the model for the collapsed data is reported (IV: ATL; DV: cycle asynchrony). This established a strong relationship between ATL and cycle asynchrony. Specifically, pairs exhibited a small degree of leading with respect to each other’s behavior at the 10 ms ATL, and increasing amounts of lagging at the 20 and 40 ms ATLs (see Figure 3A).^3^ ATL significantly predicted cycle asynchrony, *b* = 0.93, *t*(34) = 14.56, *p* < 0.001. ATL also explained a significant proportion of variance in cycle asynchrony, *R*^2^ = 0.86, *F*(1,34) = 211.88, *p* < 0.001.

### Collective Tempo

Collective tempo curves for each ATL condition illustrated characteristic patterns of tempo change over the course of a trial (see Figure 3B). On average, pairs accelerated slightly in the 10 ms condition, maintained the starting tempo in the 20 ms condition, and exhibited a substantial, progressive decrease in tempo in the 40 ms condition. An initial linear regression model for collective tempo used part similarity, unison note position, and ATL (IVs: part similarity, unison note position, and ATL; DV: collective tempo). As for cycle asynchrony, the initial model revealed no effect of part similarity and the model for the collapsed data is reported (IVs: unison note position and ATL; DV: collective tempo). This model confirmed our observations based on visual inspection of the tempo curves, with both ATL, *b* = –0.92, *t*(93) = −25.35, *p* < 0.001, and unison note position, *b* = –0.17, *t*(93) = −4.53, *p* < 0.001, significantly predicting collective tempo. This model also explained a significant proportion of variance in collective tempo, *R*^2^ = 0.88, *F*(2,93) = 331.62, *p* < 0.001.

### Note-Onset Asynchronies

We conducted a 2 (part similarity: similar, dissimilar) × 3 (ATL: 10, 20, and 40 ms) repeated measures analysis of variance (ANOVA) on the within-person difference between the frequency of leading behavior in the starter vs. joiner role (IVs: part similarity and ATL; DV: frequency of leading). This revealed no significant interactions or main effects (overall *M* = 0.32, SD = 1.1).

**FIGURE 4:**
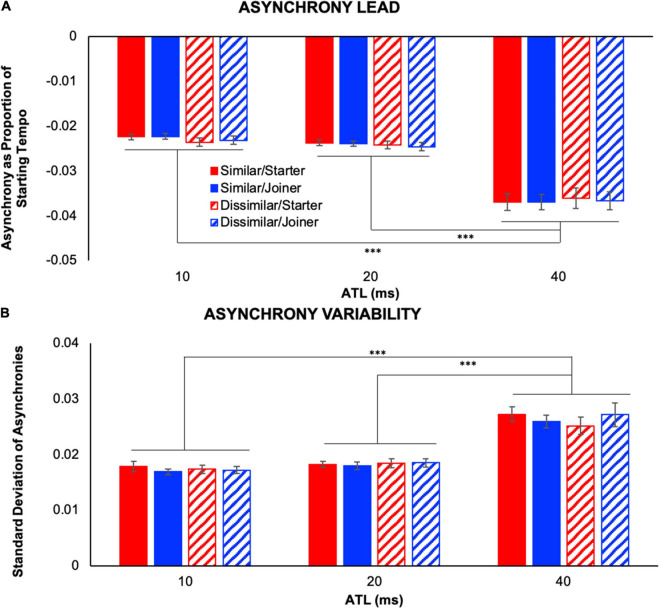
Note asynchrony between partners. **(A)** Asynchrony lead indicates the magnitude of temporal lead exhibited when the performer played first at unison note positions. **(B)** Asynchrony variability illustrates the standard deviation of asynchrony lead that occurred at unison note positions. Both measures were significantly greater in the 40 ms ATL condition than in either of the other two ATL conditions. Error bars show standard error. ^∗∗∗^*p* < 0.001.

We conducted a 2 (performer role: starter, joiner) × 2 (part similarity: similar, dissimilar) × 3 (ATL: 10, 20, and 40 ms) repeated measures ANOVA on asynchrony lead (IVs: performer role, part similarity, and ATL; DV: asynchrony lead). Specifically, this measure captured the magnitude of average temporal lead for each player when they played first at unison points (see Figure 4A). The ANOVA demonstrated a significant main effect of ATL, *F*(1.13,12.37) = 55.46, *p* < 0.001, $\eta_{p}^{2}=0.83$, but no other main effects or interactions between variables. Fisher’s LSD *post hoc* comparisons revealed a significantly larger asynchrony lead at 40 ms ATL compared to 10 and 20 ms ATL, *p*s < 0.001.

We also conducted a 2 (performer role) × 2 (part similarity) × 3 (ATL) repeated measures ANOVA on the standard deviation of asynchrony lead (IVs: performer role, part similarity, and ATL; DV: asynchrony variability). This allowed us to determine the effect of the current experimental conditions on the variability of asynchronies between performers (see Figure 4B). Like the ANOVA for average asynchrony lead, this analysis illustrated a significant main effect of ATL, *F*(1.16,12.8) = 40.86, *p* < 0.001, $\eta_{p}^{2}=0.79$, but no other main effects or interactions between variables. Fisher’s LSD *post hoc* comparisons revealed significantly larger variability at 40 ms ATL compared to 10 ms ATL and to 20 ms ATL, *p*s < 0.001.

### Individual Tempo

**FIGURE 5:**
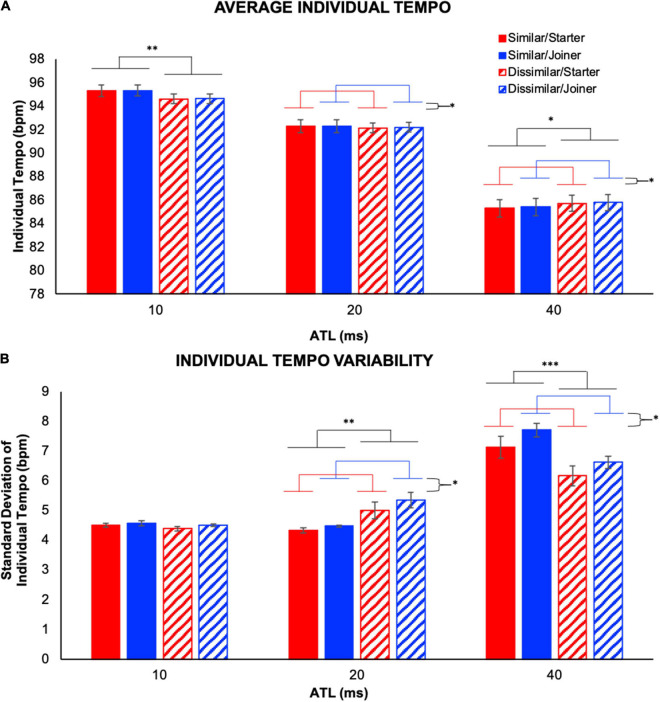
Individual temporal behavior. **(A)** Average individual tempo illustrates the tempo produced by each individual averaged over the trials. Note that the 10- and 20-ms ATL resulted in a faster tempo than the initial tempo (90 bpm on average) whereas the 40-ms ATL made performers play slower. **(B)** Individual tempo variability is computed as the standard deviation of each individual’s tempo values over the course of a trial. Significant interactions were observed between part similarity and ATL, as well as between performer role and ATL for average individual tempo **(A)**, and individual tempo variability **(B)**. Error bars show standard error. ^∗^*p* < 0.05, ^∗∗^*p* < 0.01, ^∗∗∗^*p* < 0.001.

We conducted a 2 (performer role) × 2 (part similarity) × 3 (ATL) repeated measures ANOVA on individual tempo (IVs: performer role, part similarity, and ATL; DV: individual tempo). This allowed us to identify the effect of experimental condition on performance tempo (see Figure 5A). The omnibus ANOVA revealed a significant interaction between performer role and ATL, *F*(2,22) = 5.85, *p* = 0.009, $\eta_{p}^{2}=0.35$, as well as a significant interaction between part similarity and ATL, *F*(1.22,13.45) = 10.53, *p* = 0.004, $\eta_{p}^{2}=0.49$, and significant main effects of performer role, *F*(1,11) = 7.21, *p* = 0.021, $\eta_{p}^{2}=0.40$, and ATL, *F*(1.03,11.28) = 318.40, *p* < 0.001, $\eta_{p}^{2}=0.97$. The interactions are detailed below and in Figure 5A; the main effect of performer role revealed a greater individual tempo for the joiner compared to the starter while the main effect of ATL revealed significant differences in individual tempo between all three conditions (*p*s < 0.001). The fastest individual tempo was observed at 10 ms ATL and progressively slower tempos were observed at 20 and 40 ms ATL, respectively.

To explore the interaction between performer role and ATL we collapsed the individual tempo data across the similar vs. dissimilar part similarity conditions and conducted a simple effects analysis evaluating the effect of performer role in each of the ATL conditions. We found a significant effect of performer role in the 20 ms, *F*(1,11) = 5.01, *p* = 0.047, $\eta_{p}^{2}=0.31$, and 40 ms conditions, *F*(1,11) = 8.37, *p* = 0.015, $\eta_{p}^{2}=0.43$, but not in the 10 ms condition. These analyses established that in both the 20 and 40 ms ATL conditions performers with the joiner role played faster than those with the starter role. To elucidate the interaction between part similarity and ATL we collapsed the individual tempo data across the starter vs. joiner performer role conditions and conducted a simple effects analysis evaluating the effect of part similarity in each of the ATL conditions. We found a significant effect of part similarity in the 10 ms, *F*(1,11) = 14.0, *p* = 0.003, $\eta_{p}^{2}=0.56$, and 40 ms conditions, *F*(1,11) = 8.76, *p* = 0.013, $\eta_{p}^{2}=0.44$, but not in the 20 ms condition. These analyses established that at 10 ms ATL performers played slower when their parts were dissimilar compared to when they were similar, but at 40 ms ATL the opposite was true as they played faster when their parts were dissimilar.

We also conducted a 2 (performer role) × 2 (part similarity) × 3 (ATL) repeated measures ANOVA on the standard deviation of individual tempo (IVs: performer role, part similarity, and ATL; DV: individual tempo variability). This allowed us to identify differences in individual tempo variability between the experimental conditions (see Figure 5B). The omnibus ANOVA revealed a significant interaction between performer role and ATL, *F*(2,22) = 5.22, *p* = 0.014, $\eta_{p}^{2}=0.32$, as well as a significant interaction between part similarity and ATL, *F*(2,22) = 31.86, *p* < 0.001, $\eta_{p}^{2}=0.74$, and significant main effects of performer role, *F*(1,11) = 7.85, *p* = 0.017, $\eta_{p}^{2}=0.42$, and ATL, *F*(1.27,13.96) = 70.1, *p* < 0.001, $\eta_{p}^{2}=0.86$. The interactions are detailed below and in Figure 5B; the main effect of performer role revealed greater individual tempo variability for the joiner compared to the starter while the main effect of ATL revealed significantly greater individual tempo variability in the 40 ms ATL condition than in either of the other two ATL conditions.

To further evaluate the interaction between performer role and ATL we collapsed the individual tempo variability data across the similar vs. dissimilar part similarity conditions and conducted a simple effects analysis evaluating the effect of performer role in each of the ATL conditions. We found a significant effect of performer role in the 20 ms, *F*(1,11) = 6.35, *p* = 0.028, $\eta_{p}^{2}=0.37$, and 40 ms conditions, *F*(1,11) = 7.69, *p* = 0.018, $\eta_{p}^{2}=0.41$, but not in the 10 ms condition. These analyses established that in the 20 and 40 ms ATL conditions performers in the joiner role exhibited greater individual tempo variability than performers in the starter role. To better understand the interaction between part similarity and ATL for standard deviation of individual tempo we collapsed individual tempo variability data across the starter vs. joiner performer role conditions and conducted a simple effects analysis evaluating the effect of part similarity in each of the ATL conditions. We found a significant effect of part similarity in the 20 ms, *F*(1,11) = 9.85, *p* = 0.009, $\eta_{p}^{2}=0.47$, and 40 ms conditions, *F*(1,11) = 65.58, *p* < 0.001, $\eta_{p}^{2}=0.86$, but not in the 10 ms condition. These analyses established that while there was no effect of part similarity on individual tempo variability at 10 ms ATL, at 20 ms ATL performers were more variable when their parts were dissimilar and at 40 ms ATL performers were more variable when their parts were similar.

### Perspective Taking and Locus of Control

Our results regarding the perspective taking and locus of control measures are based on a total of 23 individual participants or 11 duet pairs as one participant did not provide responses. Perspective taking scores in this group ranged from 15 to 26 (*M* = 20; SD = 3.37) and locus of control scores ranged from 22 to 41 (*M* = 32.61; SD = 5.96).

We first evaluated individual traits in relation to timing behavior. Specifically, we examined Pearson correlations between each performer’s perspective taking score and their own asynchrony lead, asynchrony variability, individual tempo, and individual tempo variability performing (1) the starter role, and (2) the joiner role (IV: performer perspective taking score; DVs: asynchrony lead as starter, asynchrony variability as starter, individual tempo as starter, individual tempo variability as starter, asynchrony lead as joiner, asynchrony variability as joiner, individual tempo as joiner, and individual tempo variability as joiner). We performed the same set of correlations for each performer’s locus of control score (IV: performer locus of control score; DVs: asynchrony lead as starter, asynchrony variability as starter, individual tempo as starter, individual tempo variability as starter, asynchrony lead as joiner, asynchrony variability as joiner, individual tempo as joiner, and individual tempo variability as joiner).

Second, we calculated the correlation between each performer’s perspective taking score and the difference in each of the asynchrony and tempo measures between the participant’s behavior when they performed the starter role vs. when they performed the joiner role (IV: performer perspective taking score; DVs: difference in asynchrony lead as starter vs. joiner, difference in asynchrony variability as starter vs. joiner, difference in individual tempo as starter vs. joiner, and difference in individual tempo variability as starter vs. joiner). We also performed the same correlations using each performer’s locus of control score (IV: performer locus of control score; DVs: difference in asynchrony lead as starter vs. joiner, difference in asynchrony variability as starter vs. joiner, difference in individual tempo as starter vs. joiner, and difference in individual tempo variability as starter vs. joiner).

Lastly, we examined possible associations between within-pair differences in co-performer trait scores and interactive behavior during performance. Specifically, we ran two additional sets of correlations. In the first set we calculated the correlation between co-performer perspective taking score differences and differences in the average starter vs. joiner behavior exhibited collectively by both co-performers for the asynchrony and tempo measures (IV: co-performer difference in perspective taking scores; DVs: difference in pair asynchrony lead for starter vs. joiner roles, difference in pair asynchrony variability for starter vs. joiner roles, difference in pair individual tempo for starter vs. joiner roles, and difference in pair individual tempo variability for starter vs. joiner roles). We did the same for co-performer locus of control score differences (IV: co-performer difference in locus of control scores; DVs: difference in pair asynchrony lead for starter vs. joiner roles, difference in pair asynchrony variability for starter vs. joiner roles, difference in pair individual tempo for starter vs. joiner roles, and difference in pair individual tempo variability for starter vs. joiner roles). In the second set of correlations involving within-pair differences in co-performer scores, we assessed the correlations between co-performer perspective-taking score differences and co-performer differences in the asynchrony and tempo measures (IV: co-performer difference in perspective taking scores; DVs: co-performer difference in asynchrony lead, co-performer difference in asynchrony variability, co-performer difference in individual tempo, and co-performer difference in individual tempo variability). We also performed equivalent correlations for locus of control scores (IV: co-performer difference in locus of control scores; DVs: co-performer difference in asynchrony lead, co-performer difference in asynchrony variability, co-performer difference in individual tempo, and co-performer difference in individual tempo variability).

**FIGURE 6:**
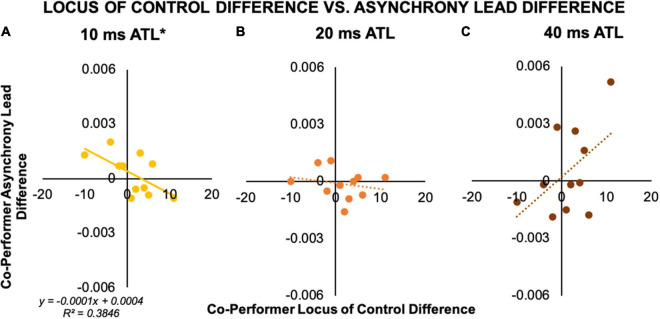
Correlation of co-performer measures between asynchrony and locus of control score. **(A) 10 ms ATL**. Within a pair, the performer with a more internal locus of control exhibited more anticipatory behavior than their co-performer in the 10 ms ATL condition. **(B) 20 ms ATL**. There was no association between within-pair differences in locus of control and asynchrony lead in the 20 ms ATL condition. **(C) 40 ms ATL**. There was a trending positive association in the 40 ms ATL condition, with relatively less anticipatory behavior by the co-performer with the more internal locus of control. *p < 0.05.

We found no correlations between perspective taking scores and any of the individual performance measures or within-pair differences. However, data from the 11 pairs we analyzed revealed an interesting pattern of preliminary correlations between within-pair differences in co-performer locus of control and co-performer differences in average asynchrony lead. Specifically, there was a significant negative correlation between co-performer locus of control difference and co-performer average asynchrony lead difference in the 10 ms ATL condition, *r*(11) = −0.62, *p* = 0.04 (*R*^2^ = 0.38; see Figure 6A). This means that the larger the trait score difference between duet partners, the larger the timing discrepancy between co-performers as reflected in the average asynchrony. While there was no association between these measures in the 20 ms ATL condition (see Figure 6B), in the 40 ms ATL condition the data trended toward a positive association, *r*(11) = 0.51, *p* = 0.11 (*R*^2^ = 0.26; see Figure 6C). As shown in Figure 6, the regression slopes (β_10_ = −0.0001, β_40_ = 0.0002) indicated similar changes in asynchrony lead difference vs. locus of control difference in the 10 and 40 ms ATL conditions, with opposite directions. Interestingly the intercepts were close to zero, showing that although this analysis was based on a relatively small number of pairs, both sets of co-performer differences made by our convenient sample of pianist pairs produced distributions around zero without skews.

## Discussion

Our findings employing piano duets are largely consistent with past work examining the effects of ATL on coordination and temporal stability during music ensemble performance (e.g., Farner et al., 2009; Chafe et al., 2010; Rottondi et al., 2015). We replicated three types of ATL effects on collective tempo such that with an ATL of 10 ms performers gradually accelerated, with an ATL of 20 ms they maintained tempo, and with an ATL of 40 ms they exhibited a progressive deceleration over the course of a trial. Our data also demonstrated that the increased asynchrony between performers at unison points was accompanied by a significant increase in the variability of asynchronies at 40 ms ATL as compared to the other two ATL conditions. This collective slowing and increased asymmetry were further confirmed in the average individual tempo and greater tempo variability. Altogether, our results demonstrate that the previously observed coordinative regime of mutual co-performer lagging and progressive tempo decline associated with ATLs at and above 40 ms is linked to a high degree of instability in both interpersonal asynchrony lead and intrapersonal performance tempo.

More importantly, however, the current study reveals novel findings on how task-related asymmetries in performer role (starting vs. joining) and musical part similarity (similar vs. dissimilar parts) shape temporal coordination, extending the replication of the overall ATL effects. Regarding performer role, it is especially remarkable that in both the 20 and 40 ms ATL conditions we saw clear differences between starters and joiners; the starters exhibited significantly lower average individual tempo and lower individual tempo variability compared to joiners. This indicates that while starters may have prioritized maintaining a stable tempo, joiners may have taken on a more adaptive role in which they adjusted their own behavior frequently to maintain coordination with their co-performer. This is consistent with the kind of functional asymmetry often exhibited by individuals assuming “leader” vs. “follower” roles in musical tasks (e.g., Fairhurst et al., 2014; Timmers et al., 2014; Chang et al., 2017) and non-musical tasks (e.g., Schmidt et al., 1994). Notably, our paradigm did not explicitly designate musical leadership to either performer. However, the starting pianist, who was given the metronome counts and started a trial as a solo, appears to have assumed leadership nonetheless. Thus, our results point to the importance of emergent leadership in temporal stability and complementary roles of musicians in duet performance.

Musical part similarity affected average individual tempo differently according to the ATL; at 10 ms ATL performers exhibited a slower average individual tempo when their parts were dissimilar compared to when they were similar. At 40 ms this was reversed such that performers displayed a faster average individual tempo when their parts were dissimilar. There was no effect of musical part similarity on average individual tempo at 20 ms ATL. This illustrates that having dissimilar parts may actually mitigate the adverse effects of ATL on individual tempo to some degree. Specifically, the acceleration observed at 10 ms ATL and the deceleration observed at 40 ms ATL appear to be somewhat diminished and there is less progressive change in tempo over time. This benefit of task complementarity is particularly interesting as previous joint action studies mostly focus on the benefits of “action simulation” for representing self and other’s action similarity through a shared coding scheme and anticipating its outcome efficiently. For example, in a joint Simon task, incongruent stimulus-response geometric mapping resulted in interference (e.g., slower reaction time) for the joint setting, and an enhanced event-related potential (ERP) component related to motor preparation (Sebanz et al., 2003; Tsai et al., 2006). Further, Novembre et al. (2014) suggested that in a piano duet task, knowledge of the other’s action represented in the motor system is key to successful coordination. This was based on the finding that TMS caused interference only for coordinating with the learned left-hand part, pre-recorded by other pianists. In our paradigm, all parts were ultimately played by all pianists, as the starting and joining roles were assigned in a counterbalanced order. This would mean that only a momentarily more active status would be given to the currently assigned duet part, compared to the partner’s part for the consecutive trials. Thus, based on the shared representation scheme, the more musically similar the parts, the more successfully the temporal coordination would have been predicted to counter the adverse effect of the ATLs on tempo drift. The opposite was observed here.

In fact, no theory accounts for how the ATL between co-actors’ actions would affect respective action representations. At least, the delayed auditory feedback for a single agent task such as auditory paced tapping is known to cause increased stimulus-tap asynchrony (e.g., Aschersleben and Prinz, 1997), indicating that theorized action planning and outcome monitoring would interact with each other. Indeed, the naturalistic delay that exists between action execution and sensory outcome is thus considered to make people assign the agency, or ownership of the movement to the outcome, and learn and calibrate the prediction according to the feedback delay (Rohde and Ernst, 2016). Within this framework, one might expect that the two action sequences assigned to self and partner would be encoded with the designated ATL. Moreover, our task employed a time-offset between the co-performers’ actions, further differentiating temporal organization between one’s own and another’s behavior. In particular, our interlocked rhythmic pattern likely assigned a momentary leadership function alternatingly to the pianist who had the solo eighth note before a given unison note, as shown in Goebl and Palmer (2009). These temporal asymmetries may play an important role in defining how action representations are managed. Especially, because the ATL was only applied to the co-performer’s sound, each performer might separately represent the other’s action *with* the uniquely associated time schedule (ATL plus rhythm-offset). When the co-performer played a musically distinct part, maintaining and monitoring the two duet parts scheduled independently in the motor system might be computationally easier than assigning two different time schedules to the shared, or similar, action sequences. The latter scenario may be prone to introducing cross-talk between self and other schedules, leading to inaccurate timing information extracted and encoded from the other’s action outcome. This view is actually compatible with another piece of our results, where at 20 ms ATL performers showed greater variability when their parts were dissimilar, and at 40 ms ATL performers showed greater variability when their parts were similar. Therefore, it is possible that performers have access to more independent timing representations of each other’s actions when their action sequences are dissimilar, pointing to the possible interaction between *what* and *when* information in joint action representation. This could then lead to decreased stability under conditions that otherwise support relatively stable coordination (i.e., 20 ms ATL) but also ultimately lead to a counter against the influence of conditions that typically perturb coordination.

Dynamical systems theory offers a mathematical framework for understanding the processes giving rise to the coordination patterns observed in our study. Here, two interacting individuals are seen as a single, multi-component system living within a “phase space” which contains all of its potential behavioral states and how they change over time (Kelso, 1995). Various symmetries and asymmetries between interacting individuals can shape these behavioral possibilities (see Richardson et al., 2016), including interpersonal social psychological asymmetries. Interestingly, past work has demonstrated that pairs of individuals with distinctly different social competence scores actually achieve more stable coordination than those with similar scores during a rhythmic synchronization task (Schmidt et al., 1994). Relatedly, pairs of individuals arbitrarily assigned to different artistic preference groups displayed greater coordination than those assigned to the same group (Miles et al., 2011). Schmidt et al. (1994) suggest that the advantage of complementarity they observed may be based on associations between the asymmetry being controlled for (e.g., social competence) and asymmetries in typical interaction behaviors such that there is a natural complementarity of stability and adaptation supporting coordination. Alternatively, Miles et al. (2011) propose that their observations may be based on a desire to lessen perceived social distance, which could also lead to increased coordinative effort. These findings point to the significance of asymmetries in determining the interplay between agents. Our current results are consistent with these past findings in demonstrating that having dissimilar musical parts is sometimes associated with greater coordination than having similar parts. Furthermore, Richardson et al. (2016) emphasize that not all types of symmetries and asymmetries consistently influence collective behavior, and that this can depend on other aspects of the interaction context. In the current study, the change in the dynamical system capturing the musicians’ interacting behavior precipitated by the introduction of different ATLs may have heightened the effect of asymmetry between co-performer parts so that at 10 and 40 ms ATL having dissimilar parts actually afforded more stable coordination than having similar parts.

We found no relationship between the self-reported perspective taking measure of empathy and any of the intrapersonal or interpersonal coordination measures. This contrasts with previous findings that individuals with higher levels of perspective taking behavior show greater adaptive behavior during rhythmic coordination (e.g., Pecenka and Keller, 2011; Washburn et al., 2019). However, those previous findings were obtained when the higher empathy was assumed to enhance the action simulation in in-person, simultaneous coordination. Thus, the discrepancy here could be explained by the above proposal with respect to possible temporal information representation required for encoding and monitoring the two complementary actions. More importantly, our results did reveal an interesting, novel pattern of potential associations between the within-pair, person-related asymmetry in locus of control scores and average asynchrony lead that appears to change as a function of the ATL. Specifically, the positive association observed at 10 ms ATL shifted to no association at 20 ms ATL, and a trending negative association at 40 ms ATL. The co-performer with a more internal locus of control may therefore have been more anticipatory than the co-performer with a more external locus of control at 10 ms ATL, but less anticipatory, and potentially more reactive, at 40 ms ATL. Because the tempo drift we observed was accelerating at 10 ms ATL and decelerating at 40 ms ATL, such individuals with the more internal locus of control may have driven the tempo drift exhibited by piano duet pairs.

With respect to the effects of co-performer differences on temporal asynchrony during coordination, Loehr and Palmer (2011) demonstrated that asynchronies between pianists were smaller when the co-performers’ individual preferred performance rates were more similar. While we did not measure individual pianists’ preferred tempo, the within-pair difference in locus of control may function in a similar manner. Notably, our findings actually indicate that within-pair difference in locus of control has a greater influence on interpersonal interaction under conditions where maintaining stable coordination is generally more difficult. Specifically, while the within-pair difference in co-performer locus of control did not impact interaction at 20 ms ATL, with the challenges to stable coordination present at 10 ms ATL and 40 ms ATL this difference did have an effect. Interestingly, our results also suggest that the individual in a pair with the more external locus of control may actually be more resilient to the coordination challenges posed by certain ATLs. This contrasts with previous findings indicating that individuals with an internal locus of control are less adaptive to the behavior of a co-performer during coordination (e.g., Fairhurst et al., 2013).

Studies with experimentally controlled musical scores allow us to observe the effects of differences in the musical structure between co-performer parts. However, in typical performance contexts such relationships between performers are likely to be dynamic, with performers exchanging who has, for example, the higher note ratio and exhibiting associated changes in temporal coordination patterns over the course of a single piece (Bishop and Goebl, 2020). Moreover, although there is evidence that performers approach etude or exercise-like material similarly to more naturalistic musical material (Brooks, 1995), it is possible that repetitive compositions lead to reduced attention or engagement with expectancy-related processes. It is therefore important that future work on NMP employ more complex musical materials as well. The incorporation of visual sensorimotor interaction within studies of NMP is also a key consideration for further study. Interestingly, Iorwerth and Knox (2019) recently illustrated that video was rarely attended by performers during NMP, despite their self-report of its importance for successful performance. In contrast, work by Hilt et al. (2020) indicates that a range of different movement kinematics sources related to ensemble performance (e.g., bow movement and head movement) each affect either inter- or intra-group coordination. Altogether, multimodal, audiovisual interaction may significantly affect the coordination of co-performers during NMP.

Notably, our evaluation of associations between individual performer personality traits and coordination during musical performance in the context of ATL is preliminary; our study was not specifically designed to include individuals with a wide range of perspective taking or locus of control characteristics or to create pairs of performers based on similarities or differences. A better understanding of how co-performer differences influence coordination, as well as other social factors related to joint action such as likeability, in contexts that involve time-shifted and asymmetric feedback such as NMP will require further targeted investigation. We also acknowledge that while our study demonstrates differences in objective measures of performance behavior in relation to ATL, we do not know what performers consciously perceived of the ATLs. Especially for the 10 ms ATL, the additional effect on top of the base apparatus latency may be not perceivable. Even with similar ensemble performance behavior there may be differences in consciousness about the effect of a delay (e.g., at the low and high ends of a range of ATLs associated with a consistent coordination regime). Other aspects of subjective performer experience in the context of NMP will also benefit from additional study. Existing work has revealed that musicians describe the physical separation associated with NMP as challenging to communication and leading to musical issues (Iorwerth and Knox, 2019). To build on this understanding, it would, for example, be valuable to investigate performer enjoyment related to ensemble coordination in the context of ATLs. Such work will be advantageously informed by the methodologies of groups like Glowinski et al. (2015), who used a combination of motion capture and semi-structured performer interviews within an Immersive Virtual Environment to identify performance strategies associated with differences in audience engagement. Relatedly, it will also be informative to gather information about the subjective experiences of audience members in relation to ATL during NMP.

Networked music performance provides an intriguing space for exploring novel composition techniques and the creation of experimental music. In an increasingly global society as well as the current COVID-19 pandemic it also provides a practical solution for musical interaction across separate geographical locations. With the need for quarantining and social distancing following the onset of the COVID-19 pandemic in early 2020 music students, educators, and professionals have sought opportunities to continue learning, rehearsing, and performing together. This has driven rapid and transformative improvements in the primary platform for NMP, a multi-machine technology called JackTrip which supports bidirectional flows of uncompressed audio over the internet at the lowest possible latency (JackTrip, 2021). Advancements have focused on ease of use and scaling across worldwide cloud infrastructure to support a range of activities, including rehearsal for orchestral-sized ensembles. This ongoing development led by developers and musical practitioners in conjunction with the recently established JackTrip Foundation constitutes a significant contribution to the potential utility of NMP across contexts (JackTrip Foundation, 2021). Other researchers have also proposed a global metronome for facilitating NMP (Oda and Fiebrink, 2016; Hupke et al., 2019a,b). Most recently this has included the presentation of an adaptive metronome capable of supporting increased synchronicity at higher delays and reducing tempo drift *via* a low-latency solution for when high-end hardware is not available (Battello et al., 2020). Collectively, these efforts are generating technologies that will increase the quality and accessibility of cyber-mediated musical interaction far beyond the needs of the current pandemic. The existence of functional, remote musical education, for example, would greatly increase the availability and frequency of music education activities worldwide.

Our study directly informs how musical interaction in NMP could be designed with the presence of the ATL. Notably, our findings indicate that ATLs around 20 ms are most likely to support stable coordination. Internet connections supporting ATLs of 20 ms or lower will therefore be more appropriate for the majority of NMP applications than those with longer latencies. Interestingly, we also observed that task-related asymmetries, such as dissimilarity between musical parts, may increase the coordinative stability between co-performers. Accordingly, individuals leading music education activities *via* NMP might aim to prioritize exercises involving complementary, asymmetric musical tasks, especially in cases where ATL is variable or cannot be kept to a minimum around 20 ms. Further experimental research into the effects of ATL on interpersonal coordination during musical performance, including the ongoing development of computational models capturing multi-agent coordination in the context of informational delays (e.g., Rottondi et al., 2016; Demos et al., 2019; Heggli et al., 2019; Román et al., 2019; Shahal et al., 2020), is invaluable to the continued improvement of NMP. Performers have also indicated that physical separation alone poses challenges to music ensemble performance independent of those introduced by ATLs, noting that the associated hindrances to tuning, blending, and taking musical risks can all inhibit creativity (Iorwerth and Knox, 2019). The significant and transformative implication for cyber-interaction is that together researchers, developers, and performers have the opportunity to: (1) understand the effects of physical separation and perceptual-motor delays on complex musical interaction, and (2) use this information to shape the evolution of technologies for robust, versatile, and rewarding NMP.

## Data Availability Statement

The raw data supporting the conclusions of this article will be made available by the authors, without undue reservation.

## Ethics Statement

The studies involving human participants were reviewed and approved by the Stanford University Institutional Review Board. The patients/participants provided their written informed consent to participate in this study.

## Author Contributions

MW created the experimental apparatus and MW and AW verified its setup. AW and MW collected the data. AW and TF analyzed the data and wrote the manuscript. All authors designed the study and edited the manuscript.

## Conflict of Interest

CC was employed by the company JackTrip Labs, Inc. The remaining authors declare that the research was conducted in the absence of any commercial or financial relationships that could be construed as a potential conflict of interest.

## Publisher’s Note

All claims expressed in this article are solely those of the authors and do not necessarily represent those of their affiliated organizations, or those of the publisher, the editors and the reviewers. Any product that may be evaluated in this article, or claim that may be made by its manufacturer, is not guaranteed or endorsed by the publisher.

## Footnotes

1 One participant reported a hearing impairment at high frequencies necessitating the use of a hearing aid in a classroom but not while playing music.

2 The speed of sound in air is 343 m/s, at 68°F (20°C) and 1 atmosphere pressure. For example, a distance of 4 m would produce 11.6 ms delay.

3 We are presenting cycle asynchrony and note-onset asynchrony results following the simple sign. This means that the individual who plays their note first during a given note-onset asynchrony will have a smaller time value than the individual who plays second, resulting in a negative value associated with leading behavior during asynchrony. This approach is consistent with many coordination researchers but is the opposite of how results were presented by Chafe et al. (2010). Ultimately this difference in approach does not affect the interpretation of the results.
